# How much calcium to shell out? Eggshell calcium carbonate content is greater in birds with thinner shells, larger clutches and longer lifespans

**DOI:** 10.1098/rsif.2021.0502

**Published:** 2021-09-29

**Authors:** Stephanie C. McClelland, Phillip Cassey, Golo Maurer, Mark E. Hauber, Steven J. Portugal

**Affiliations:** ^1^ Department of Biological Sciences, School of Life and Environmental Sciences, Royal Holloway University of London, Egham TW20 0EX, UK; ^2^ Invasion Science & Wildlife Ecology Lab, University of Adelaide, Adelaide, South Australia 5005, Australia; ^3^ BirdLife Australia, 2/5, 60 Leicester Street, Carlton, Victoria 3053, Australia; ^4^ Centre for Tropical Environmental and Sustainability Studies, College of Science and Engineering, James Cook University, Cairns, Queensland 4878, Australia; ^5^ Department of Evolution, Ecology, and Behavior, University of Illinois at Urbana-Champaign, Urbana, IL 61801, USA; ^6^ The Natural History Museum, Tring HP23 6AP, UK

**Keywords:** avian, clutch size, eggs, investment, life history, phylogenetics

## Abstract

The avian eggshell is a bio-ceramic structure that protects the embryo. It is composed almost entirely of calcium carbonate and a small amount of organic material. An optimal amount of calcium carbonate in the eggshell is essential for the embryo's development, yet how the ratio of calcium carbonate to organic matter varies between species has not been investigated. Calcium is a limiting resource for most birds, so its investment in their eggs should be optimized for a bird's life history. We measured the relative calcium carbonate content of eggshells in 222 bird species and tested hypotheses for how this trait has evolved with the life-history strategies of these species and other traits of their respective egg physiologies. We found that (i) eggshell calcium carbonate content was positively correlated with species having thinner eggshells and smaller than expected eggs relative to incubating parental mass, (ii) species with small mean clutch sizes had lower calcium carbonate content in their eggshells, and (iii) for species with larger clutch sizes, eggshell calcium carbonate content was negatively correlated with their mean lifespan. The pattern of lower eggshell calcium carbonate in longer lived, larger clutched birds suggests that calcium provision to the eggshell has long-term costs for the individual.

## 1. Introduction

Life-history theory explains what determines when, how and to what extent reproduction should occur for an organism to optimize its individual fitness [1]. A key aspect of these reproductive strategies is an investment in individual reproductive bouts versus self-maintenance, and the spreading of investment over multiple reproduction attempts [1,2]. The avian eggshell, as an extension of both a bird's phenotype and its life history, is under the influence of strong selective factors, since embryonic development and reproductive success are highly dependent on the optimal functionality of the eggshell [3,4]. Birds' eggshells have evolved many specific adaptations in their composition and structure for ensuring successful embryonic development across different life histories, nest environments and climatic conditions [5,6]. Egg production provides a critical example of life-history theory in action as the investment into an egg and/or clutch will greatly influence the quality of that offspring, but, conversely, will reduce the parent's resources for both immediate self-maintenance and future reproductive investment [7]. This trade-off has been explored in the context of egg contents [8], such as androgen deposition in the yolk [9,10] and pigment deposition in the shell matrix [11], yet the production of the eggshell itself and its composition have not been considered within the same framework.

The avian eggshell performs multiple functions to enable and facilitate embryonic development. The eggshell provides a rigid armour to protect the developing embryo from mechanical damage and acts as a physical barrier to microbial infection [12]. Moreover, the eggshell controls the appropriate exchange of heat, water and respiratory gases with the immediate nest environment [13], while also providing a reservoir of calcium and other trace minerals for absorption by the developing embryo [14]. Simultaneously, pigment deposited on the outer surface can play an important role in varied behaviours, such as crypsis, thermoregulation and sexual signalling [15–18]. The evolution and adaptations of the eggshell have allowed birds to breed in almost all terrestrial environments and habitats globally [19]. A key component of this success has been the presence of calcium carbonate in the eggshell in the form of calcite [4,20]. How calcite crystals form to produce the structure of the eggshell has been rigorously studied [21,22], and the detrimental impacts of calcium deficiency on reproduction are well established [23,24]. Despite this, the quantity of calcium carbonate in the shell has rarely been considered as an evolved trait in bird species (but see [25]), even though broad-scale macro-ecological studies have found global patterns in egg shape [26], egg size [27,28] and shell pigmentation [29–31].

Eggshells are sophisticated bio-ceramic structures consisting of a calcium-based mineral structure interwoven with an organic protein matrix [12,32,33]. Calcium carbonate is believed to make up approximately 98% of the eggshell for most bird species [16,34], though the variation across species has not been previously explored. An appropriate amount of calcium carbonate deposited in the eggshell is essential for the embryo to develop correctly, as incomplete calcification of the shell can lead to overly large pores and desiccation, while excess calcium can lead to severely reduced gas exchange [34,35]. Insufficient calcium in the shell can also cause the embryo to become hypocalcaemic, resulting in retarded growth or, in extreme cases, death [36,37].

Here, we investigate the macro-phylogenetic patterns present in eggshell calcium carbonate content across a large number of diverse avian species and investigate the relationship between eggshell calcium carbonate to organic component ratio and a species' life-history traits. Many life-history traits can be expected to impose constraints or trade-offs in the amount of calcium allocated to the eggshell. Calcite or its isoforms cannot be stored to any significant amounts in most avian bodies [34,38], though cyclic osteoporosis can provide a portion of the calcium for egg formation in some species [39]. As such, this mineral must be obtained from the mother's diet during egg formation [34]. Acquiring sufficient calcium for egg production for many species requires behavioural adaptations such as diet switching and/or strenuous foraging beyond their normal requirements and outside their normal ranges, potentially increasing inter-territorial disputes [40,41]. It is assumed that the greater the number of eggs produced, the less calcium available to be provisioned to each [42].

The structure of the shell is under differing selective pressures to optimize strength, gas exchange and hatchability [3,5], among other factors, each of which might cause contradicting directional selection on the eggshell calcium carbonate content. We considered a number of pertinent life-history traits where there is evidence of selection on other aspects of egg physiology and formulated 10 key hypotheses and predictions with respect to the eggshell calcium content in 222 species (table 1). These hypotheses were subdivided based on the framework of Tinbergen's four questions to address variation in eggshell calcium carbonate content between species from a mechanistic, proximate perspective (mechanism and ontogeny) and from a broader adaptive, evolutionary perspective (adaptation and phylogeny) [43,44]. The goal of our novel investigation into macro-evolutionary patterns of a key eggshell trait was to explore new associations between eggshell content and avian life history, phylogeny and physiology.

**Table 1.** Hypotheses and predictions with supporting rationale of how eggshell calcium carbonate content in birds relates to life-history strategies and eggshell characteristics. Hypotheses are divided based on Tinbergen's four-question structure [43,44].

level of question/prediction	hypothesis	prediction	rationale and/or proposed mechanism
mechanism	1) Thicker eggshells are achieved through greater deposition of calcite but not matrix during layer formation, resulting in higher relative calcium carbonate content of thicker eggshells.	Species with eggs that have thicker shells also produce shells with higher calcium carbonate content than species with thin-shelled eggs.	The crystalline structure of the shell is believed to be controlled primarily by the organic matrix, which modulates the deposition of calcium from the uterine fluid [45,46]. Selection for thicker eggshell could increase the binding of calcite crystals to the organic matrix during shell formation.
	2) Calcium carbonate content of eggshells is influenced by diet.	Species with diets that are normally higher in calcium invest more calcium in their eggshells.	The majority of calcium needed for egg production must be obtained from their diet during egg formation [34].
	3) Eggshell pigmentation has evolved to compensate for lower calcium carbonate content.	Pigmented eggshells contain less calcium carbonate than immaculate eggshells.	In great tits (*Parus major*) and Eurasian sparrowhawks (*Accipiter nisus*) calcium stress and eggshell thinning have been correlated with more pigmented eggshells, suggesting protoporphyrin pigment might be used to strengthen eggs in compensation for lacking calcium [47,48].
			However, in another species (black-headed gulls; *Larus ridibundus*) the correlation between pigmentation and shell thinning was found to be weak [49].
	4) Species eggshell calcium carbonate content is adjusted to their breeding latitude as a result of calcium availability and selection for thicker shells in colder climates.	Species breeding at higher latitudes (further from the Equator) will have a higher calcium carbonate content in their eggs.	Multiple egg traits are known to vary latitudinally at both an inter- and intraspecies level, believed to be a response to variation in temperature and solar radiation [31,50,51]. There is evidence that thicker eggshell can retain heat longer, which may benefit species breeding at colder latitudes [52], which led to greater calcium carbonate content in these eggs. Additionally, calcium availability in the environment is known to increase in higher latitudes [42].
ontogeny/proximate	5) Precocial species deposit more calcium overall into their eggshell in order to supply the higher demand for embryonic growth without compromising the integrity of the eggshell through excessive thinning.	Eggshell calcium carbonate content is higher in species with precocial modes of development.	Nestlings of precocial species hatch in a more developed state than those of altricial species, in particular they have a more ossified skeleton and muscles and larger brains [53]. This requires greater sequestration of calcium during development, which is supplied by a greater number of mammillary tips of the eggshell [15,53].
	6) Incubation period influences calcium carbonate content.	Species with longer incubation periods will have more calcium carbonate in their eggshell.	Longer incubation period requires less porous eggshells to prevent excessive water loss, and as a result may have denser eggshell produced through greater calcite crystal deposition [54,55], showing an evolutionary relationship between eggshell porosity and incubation length in Alcidae species.
adaptation/ ultimate	7) Calcium carbonate content is influenced by reproductive investment (clutch size).	Calcium carbonate content decreases with increasing clutch size	Patten [42] suggested that the evolution of clutch size is influenced by the availability of calcium in the breeding habitat. This would suggest a strong correlation between clutch size and eggshell calcium content.
	8) A species lifespan influences calcium carbonate content per egg.	Lifespan is negatively correlated with calcium carbonate content.	If calcium foraging is an expensive activity, longer lived species might invest less calcium in eggs per clutch in order to conserve energy for future reproductive attempts compared with species which only have the opportunity to breed a few times over their short lifespan. There is evidence that lifespan influences egg size and clutch size in birds [56].
	9) Eggshell calcium carbonate content is higher in species with eggs that are smaller than predicted for the mass of the incubating parents.	Calcium carbonate content will be predicted by the residual difference between fresh egg weight (as a proxy for egg size) and adult body mass.	Egg traits such as the size, shape and thickness of eggs have evolved in tight concert with adult body mass, as the egg needs to be able to support the weight of the parent during incubation yet remain thin enough to allow the chick to hatch [3,57]. Smaller eggs experience a greater force per unit area of the shell from the mass of the incubating parent and as such could require a higher calcium carbonate content to compensate.
phylogeny/ultimate	10) A large component of variation in eggshell calcium carbonate content is correlated with the species' phylogenetic position.	Calcium carbonate content has a phylogenetic signal close to, but less than, 1 (Pagel's *λ*) [58].	Many eggshell characteristics have been shown to strongly covary with phylogenetic relatedness in birds [5,30,59]; as such we expect eggshell calcium carbonate content to be similarly correlated to phylogeny.

## 2. Methods

### 2.1. Calcium carbonate content (ash) of eggs

All eggshells were obtained from the Destructible Collection at the Natural History Museum, Tring, UK, a unique resource containing blown eggs mainly of European breeding birds, identified to species levels but otherwise too data-poor to allow admission to the museum's main collection [5,30]. Owing to limitations of the information available for this (destructible) subset of the collection, we did not have specific details about where eggs were collected or the clutch size they were taken from. Eggs were assumed to be freshly laid at collection because of the small size of blow holes. A small blow hole suggests no substantial embryo was present, as the liquid egg content could be extruded through this narrow opening. All eggshells were cut in half vertically (from sharp to blunt pole) using a diamond-tipped dentist drill (Milnes Bros., Croydon, UK). One half of each egg was weighed on a precision electronic balance (Sartorius, Göttingen, Germany), before being put in an oven at 60°C to dry to a constant mass. To assess this, all shell halves were weighed individually twice daily, between 09.00–10.00 and 16.00–17.00, until no change in mass was detected for four consecutive weighing sessions, at which point they were considered ‘dry’. Following this, each shell half was placed in a small ceramic crucible and weighed with this container. The crucibles with the dry shell were then placed into a muffle furnace (AAF 1100; Carbolite, Hope, UK) for 30 h at 650°C to burn off the organic component of the shell. Immediately after removal from the furnace, each crucible with the shell ash was placed in a desiccator to cool down without absorbing moisture from the air before being weighed again. Calcium carbonate content was calculated as the ash mass of the shell half, as a percentage of the dry mass of the shell half. Other inorganic minerals that occur in trace amounts alongside calcium carbonate in the eggshell (e.g. phosphorus and magnesium) were not considered separately as they occur in extremely small quantities (less than 0.1% of the eggshell) [60,61]

### 2.2. Life-history and physical egg traits

Life-history and ecological data were gathered primarily from the *Handbook of the Birds of the World*, volumes 1–13 [62], and cross-referenced with birds of the Western Palaearctic [63]. Body mass of adult birds was taken as a mean of the mass of both sexes, primarily from the *Handbook of Avian Body Masses* [64]. The residual variation in egg size was calculated as the residual variance of each species from the predicted values of a linear correlation between log_10_ corrected body mass and log_10_ corrected fresh egg mass. Lifespan was extracted from [65], and mean breeding latitude was calculated from [66]. Clutch size data were collected as a mean number of eggs but subsequently divided into two categories, with species producing either a single egg or two eggs per clutch categorized as ‘small’ and all other species categorized as ‘large’. This is because of an unequal distribution of clutch sizes in the data (electronic supplementary material, figure S1) and preliminary results supporting a categorical rather than a continuous effect of clutch size. Species mean eggshell thickness values were extracted from [17].

### 2.3. Statistical analyses

All statistical analyses were conducted in R statistical software (R v. 3.3.2 [67]) through the Integrated Development Environment ‘R Studio’ [68]. A phylogenetic tree was constructed for the 222 species included in this study from the Open Tree of Life project, using the R package ‘rotl’ [69], which constructs a tree using multiple taxonomies as a backbone. The strength of the phylogenetic signal (Pagel's *λ*) in the calcium percentage of the eggshells was estimated on the mean values for each species, using the ‘phylosig’ function in the R package ‘phytools’ [70,71]. The R package ‘caper’ [72] was used to construct phylogenetically informed least-squares (PGLS) models using the constructed phylogenetic tree. In these models, we were able to include phylogeny and Pagel's *λ* as a covariance matrix, thereby accounting for phylogenetic non-independence of the residual error in the response variable (calcium content). Pagel's *λ* was assigned by maximum likelihood in all models [73].

Calcium carbonate percentage was first arcsine transformed to account for the proportional nature of the data and then log_10_ transformed to account for a non-normal distribution (electronic supplementary material, figure S2). The resulting metric was our response value in the subsequent models and was tested against life-history and physiological traits as predictors. PGLS models require a single response value per species; as such mean calcium carbonate content was determined for each species. To test our hypotheses, candidate PGLS models [71] were constructed with combinations of the following predictors: log eggshell thickness (mm), residual egg size variance relative to adult body mass (g), precociality—assigned categorically by whether or not eyes are open at hatching (precocial/altricial), mean clutch size (small (not more than 2) or large (2.5–16)), mean incubation period (days), species mean breeding latitude (degrees), species diet (omnivore or carnivore; no herbivores were available in the dataset), mean lifespan (years) and whether eggs are pigmented or immaculate (yes/no). Several two-way interactions were also included in PGLS models, listed here (*denotes interaction): log eggshell thickness*calcium diet, log eggshell thickness*precociality, log eggshell thickness*clutch size, lifespan*clutch size, clutch size*precociality, mean incubation period*clutch size, mean incubation period*precociality and lifespan*precociality.

These candidate models were ranked based on Akaike information criterion values corrected for small sample sizes (AICc), and model averaging was applied to all models (*n* = 3), which could not be rejected based on having an AICc score within two points of the lowest AICc-valued model [74]. The R software package ‘MuMIn’ was used for model selection and averaging [75]. The averaged model produced contained only the predictors: log eggshell thickness, residual egg mass, lifespan, clutch size, latitude, and the interaction between lifespan and clutch size (electronic supplementary material, table S1).

**Figure 1.**
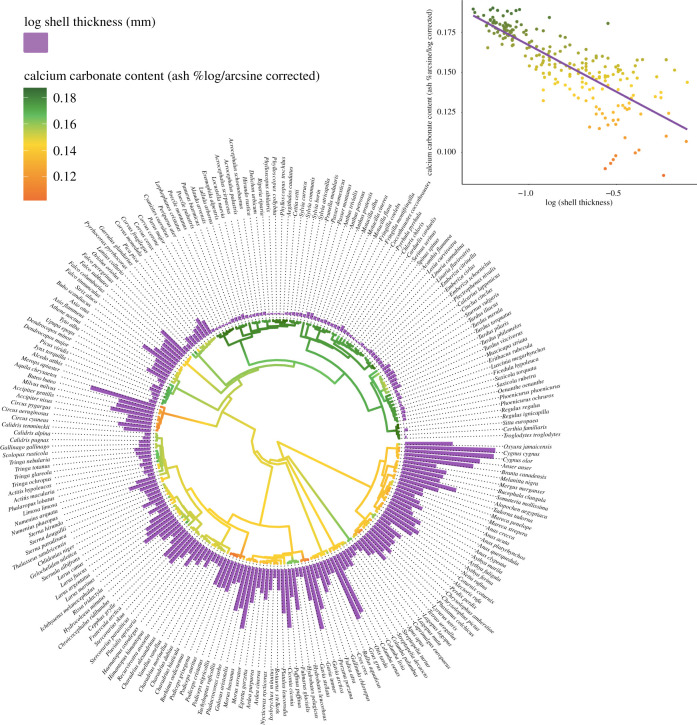
Phylogenetic tree of mean eggshell calcium carbonate content (ash % of dry eggshell mass) of species' eggs. Phylogenetic tree of all included species (*n* = 222) generated from the Open Tree of Life [69]. Branch colour represents an ancestral reconstruction of eggshell calcium content (log arcsine of eggshell calcium %) with green representing higher calcium carbonate content and orange representing a lower content. Purple bars display log eggshell thickness (mm) of each species. Inset graph: calcium carbonate content (ash % of dry eggshell mass) predicted by (log) eggshell thickness.

PGLS models can only compare mean calcium content value [76] and do not account for intraspecies variability; to account for this, we further constructed a phylogenetically informed multivariate mixed model (PMM) [77], which included all measurements per species (samples per species varied between *N* = 1 and 5; see electronic supplementary material, table S3), tested against the predictors of the averaged PGLS model listed above. The PMM was fitted with the package ‘sommer’ R v. 4.0 [78], using the same phylogenetic tree described above. The phylogenetic tree (figure 1) was visualized using the ‘ggtree’ package [79].

## 3. Results

Our final PMM (containing predictors identified by model averaging of PGLS candidate models; electronic supplementary material, table S1) contained the predictors log shell thickness and residual egg size variance, as well as key life-history traits of clutch size, lifespan, the interaction between clutch size and lifespan, and mean breeding latitude. All other predictors and interactions were not retained in the averaged model set of PGLS models, indicating that these variables neither improved the fit of the model nor were significant predictors of eggshell calcium content, and as such were not included in the PMM. There was an effect of phylogeny on mean eggshell calcium carbonate content with an intermediate Pagel's *λ* value of 0.82, which was significantly different from both zero and 1 (*p* > 0.005, 95% CI: 0.686, 0.906), suggesting close relatives were correlated in the values of eggshell calcium content, though less than would be seen under a strict Brownian motion model of evolution.

**Figure 2.**
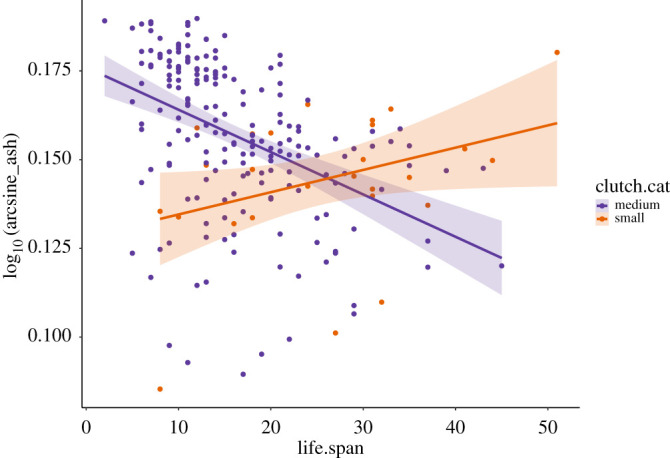
Mean carbonate calcium content (ash % of dry eggshell mass) of species' eggs in relation to lifespan (in years) and clutch size (eggs/nest). Mean eggshell calcium carbonate content of 222 species (log – arcsine transformed) calculated from 817 eggs, showing ash percentage decreases with increasing lifespan in species with large-sized clutches, but not species with small clutches (*t* = 3.13, *p* = 0.002). The regression lines are representative of linear regression, not corrected for phylogenetic relatedness.

Calcium carbonate content was negatively correlated with an eggshell thickness (estimate = −0.04, s.e. ± 0.006, *t* = 6.86, *p* < 0.005), after accounting for phylogeny, with thicker eggshells having a lower calcium carbonate content (as a percentage of dried shell mass) than thinner eggshells (figure 1). There was a significant effect of the residual variation in adult body mass relative to egg mass on eggshell calcium carbonate content (estimate = −0.01, s.e. ± 0.005, *t* = 2.46, *p* = 0.01), indicating that species with eggs that were larger than expected for their adult body mass had a higher eggshell calcium carbonate content. Calcium carbonate content was also predicted by clutch size, with species with smaller clutches having lower eggshell calcium carbonate content (estimate = −0.02, s.e. ± 0.007, *t* = −2.85, *p* = 0.004). Additionally, there was an interaction between clutch size and lifespan on calcium carbonate content (figure 2): among species with a clutch size over two eggs, calcium carbonate content of eggs decreased with increased lifespan; however, this effect was not evident in species with less than an average of 2.5 eggs per clutch (interaction, estimate = 0.0007, s.e. ± 0.0002, *t* = 3.13, *p* = 0.002). There was also a pattern of lower eggshell calcium carbonate content at higher breeding latitudes (estimate = 0.0001, s.e. ± 0.00005, *t* = 2.63, *p* = 0.012). Lifespan alone was not a significant predictor of eggshell calcium carbonate content (*p* = 0.99) outside of the interaction with clutch size. The high value of phylogenetic signal (*H*^2^ = 0.80 ± 0.04) of the PMM (accounting for intraspecific variation) was consistent with the high Pagel's *λ* value found for mean calcium carbonate content.

## 4. Discussion

Our results support several of the proposed hypotheses, such as a species lifespan and clutch size dictating its eggshell calcium investment, while also showing an interesting negative correlation with eggshell thickness, which was opposite to our predictions (electronic supplementary material, table S2). We found that differences observed in eggshell calcium carbonate content covary with a combination of physiological traits (eggshell thickness and egg mass) and life-history traits (lifespan, clutch size and breeding latitude). We found a phylogenetic signal in the variation in eggshell calcium carbonate content between species that was stronger than would be expected if this trait was evolving neutrally (Brownian motion model of evolution) [58], meaning that closely related species were more similar to one another than distantly related species, as a result of shared ancestry [73]. This would suggest that calcium carbonate content is under strong genetic control, as is the case for other known calcium-related eggshell properties such as calcite crystal size and organization [80].

Some of our results—lower eggshell calcium carbonate content in longer lived, large clutched species—indicate that the allocation of calcium in avian eggshell production is likely to be a feature of life-history evolution to maximize lifetime fitness. These findings complement current understandings of life-history evolution [1], assuming calcium deposition in eggshells is costly to the female [34,81]. Species with shorter lifespans are likely to have fewer opportunities to reproduce and, as such, are more likely to invest heavily in the few broods that they do produce [1]. By contrast, long-lived species may reserve energy and resources for future reproduction at the expense of their current reproductive effort [7]. Excess calcium is not known to be stored in the body long-term in most birds, meaning that the current investment of calcium into a brood is unlikely to significantly impact future calcium availability [34]. However, the investment of calcium into a clutch of eggs may have other costs to future reproduction. The calcium needed for egg production must be acquired from the environment within a brief window prior to egg laying, in order to increase circulating calcium [34,38,81]. This requires strenuous foraging, often for food sources that differ from the usual diet or requiring extraterritorial excursions, which increases energy expenditure and predation risk for the female [40,41,82]. The extent of calcium-targeted foraging can have an impact on body condition and, therefore, probability of survival to the next breeding season [83]. Females of many bird species are believed to be osteoporotic during egg laying as a result of calcium sequestration from medullary bones [34], especially where dietary calcium is limited [39], resulting in higher susceptibility to skeletal fractures [84]. Reducing the calcium carbonate content of eggshells might, therefore, present a trade-off between producing eggs with a strong shell and bountiful calcium supplies for the embryo, or optimizing lifetime reproductive output by producing many clutches of eggs with sufficient but less than ideal eggshell calcium carbonate content.

For bird species with small clutches (one or two eggs), there was no statistical effect of lifespan on eggshell calcium carbonate content. Overall, species with small clutch sizes had lower calcium carbonate content per eggshell than other birds. Investment strategies of species producing such ‘micro-clutches’ might differ from the investment strategies predicted in larger clutched birds [85,86]. One theory of clutch size evolution is that a greater risk of predation selects for smaller clutches [28,87]. As small clutches are associated with species under high predation risk [28,88], it would be strategic to reduce the calcium carbonate content of these eggs, in addition to reducing clutch size, in favour of survival and conserving body condition for future reproductive attempts by the female. This would especially be the case if calcium foraging increases the risk of adult mortality by increasing predation risk, as has been proposed but not tested [41].

There is a global gradient of increasing environmental calcium availability with higher latitude, which is thought to have influenced the evolution of bigger clutches at higher latitudes [42]. We expected to see higher calcium carbonate content in eggs of birds breeding at higher latitudes owing to this greater availability, and potential selection for denser shells in colder climates. However, contrary to this, we found a decrease in proportional calcium carbonate content in eggshells of species breeding at higher latitudes. As this study is composed primarily of species breeding in the North Hemisphere, increasing latitude corresponded to a greater distance from the Equator. Although this does not correspond with global calcium availability patterns, or our rationale regarding temperature, there are many other factors that vary latitudinally, such as climate and food availability [89,90], and as such it is difficult to identify the root cause of latitudinal variation. Additionally, the present study relied on mean breeding latitudes of these species, as detailed information on collection location did not exist for these eggs. As such, we were unable to account for intraspecific variation in latitude. Future studies should consider intraspecific variation and compare high-latitude, temperate species with those endemic to the tropics where environmental calcium availability is dramatically lower [42].

In addition to correlations with life-history traits, there was a strong negative pattern between species' eggshell thickness and eggshell calcium carbonate content. This is likely to be linked to the strength requirements of the eggshell, which needs to be finely balanced between being strong enough to support the body mass of the incubating parent while also remaining breakable from the inside for the chick to hatch [3,91]. Eggshell strength increases with eggshell thickness [91], although other factors such as egg shape or calcite crystal size and orientation also influence strength [57,92,93]. However, our results indicate that the increased strength with increasing thickness may not be achieved through greater calcium carbonate deposition, but rather a thicker eggshell may achieve this greater strength via alternative mechanisms. The eggshell is formed by the precipitation of calcium carbonate from the uterine fluid to form calcite crystals on the surface of the egg membrane [12]. The formation of these crystals, particularly the unit size of each crystal and how they orientate to and interlock with each other, is controlled by the organic component of the eggshell [12,92]. Moreover, this is highly heritable [80] and largely determines the strength of the shell [93,94]. An increase in osteopontin, a major component of the organic portion of the shell, leads to smaller crystal units in the nanostructure of the shell, which increases the overall hardness of the material [92,95]. Additionally, the binding of osteopontin to calcite crystals during formation increases fracture resistance [96]. The observed lower calcium carbonate content in thicker shelled eggs indicates a greater organic component, which could strengthen the shell in such a manner [92]. Further investigation into how calcium carbonate content directly correlates with fracture resistance would be useful to elucidate this. Lower calcium carbonate in thicker eggshells may be a constraint of other required properties of the shell, such as flexibility and stiffness, which will vary with allometric scaling and thickness [57]. Conversely, a thinner eggshell might require more calcium carbonate to be formed into denser calcite crystals to be strong enough to protect the egg. Eggshell thickness and egg size are strongly and positively correlated [3,97]; as a result, it is feasible that, in smaller eggs, an increase in thickness would increase the required interior breaking force (difficulty for the chick to hatch) to a greater extent than for larger eggs, owing to shape and allometry [57,91,97]. As such, smaller eggs may achieve strength through denser calcium carbonate deposition while remaining thin enough for the developed chick to hatch. Further investigation into the role of calcium carbonate content in the structural properties of eggshells would be beneficial to our understanding of how this trait has evolved. Potentially, this association between eggshell thickness, calcium carbonate and size could explain the low eggshell calcium carbonate content seen in small clutches, since eggshells of single-egg clutches tend to be larger and hence thicker shelled [3,13,91,97].

There is a consistent scaling relationship between egg size, eggshell thickness and the body mass of the incubating parents [3,91]. We found that eggshell calcium carbonate content decreases as species' residual body mass (body mass relative to egg mass) increases. As such, species with eggs that are small relative to the size of the incubating parent have a lower calcium carbonate content in their eggshells, which would suggest that the shell's ability to support the mass of the incubating parent is not increased with calcium carbonate content. This agrees with the above discussion that a greater organic component could imbue greater strength to eggshells by regulating the organization of calcite crystals [92,95]. Additionally, high calcium carbonate content in eggs that are larger than predicted for a species' body size would probably represent a substantial investment. Body mass is tightly positively correlated with skeletal mass in birds [98] and will likewise affect the potential quantity of circulating blood calcium that can be maintained during egg production, thereby increasing the rate at which calcium must be obtained during the period of shell formation [34,82,99]. This is relevant to our understanding of the costs of egg production and how they affect investment strategies across avian families.

Our findings change the understanding of how avian species allocate mineral resources to their eggs and how this connects with their life-history investment strategies. Calcium in eggs has long been acknowledged as an important factor for reproductive success; however, the association with lifespan should make us reconsider the investment costs involved. Along with the strong phylogenetic signal, this suggests that species are under selection to optimize individual per egg calcium allocation for maximum lifetime reproductive success. This contradicts previous suggestions that calcium allocation to eggshells does not apply a long-term cost to breeding females [100]. These findings highlight how little we know about the costs associated with calcium acquisition, and what the benefits are to the eggshell's structural integrity of a higher or lower calcium carbonate content. Additionally, it is not yet known what genetic factors control calcium allocation during eggshell formation and how flexible this trait is within a species under different conditions. Eggshell thinning as a result of environmental pollution [101,102], but also reduced environmental calcium availability [103,104], has had severe detrimental effects on bird populations. A greater understanding of the optimal eggshell composition for a species' reproductive biology and life history would enable us to better assist breeding programmes for endangered birds. The specialization in shape and microstructure of eggshells has evolved these vessels to be highly optimized for embryo development given a species' specificities [5,26], and these results show how eggshell calcium content has likewise evolved to complement avian life histories.

## Data accessibility

Additional data are provided in the electronic supplementary material [105] and at 10.6084/m9.figshare.16560003 and 10.6084/m9.figshare.16559985.
